# Comparative safety of the 20-valent and the 13-valent pneumococcal vaccines in infants and toddlers: a systematic review and meta-analysis of randomized trials

**DOI:** 10.1590/1984-0462/2026/44/2025047

**Published:** 2026-11-09

**Authors:** Helena Beckhäuser Ubaldo, Maria Clara Abreu Vianna Braga, Elisama da Costa Silva, Lenita de Melo Lima

**Affiliations:** a Universidade Federal de Santa Catarina, Florianópolis, SC, Brazil.; b Universidade Salvador, Salvador, BA, Brazil.; c Instituto Universitario de Ciencias de la Salud, Fundación Héctor Alejandro Barceló, Buenos Aires, Argentina.; d Universidade Estadual do Mato Grosso do Sul, Campo Grande, MS, Brazil.

**Keywords:** Pneumococcal vaccines, Vaccine safety, Adverse event, Systematic review, Meta-analysis, Vacinas pneumocócicas, Segurança vacinal, Evento adverso, Revisão sistemática, Metanálise

## Abstract

**Objective:**

This systematic review and meta-analysis aimed to compare the safety of the 20-valent pneumococcal conjugate vaccine versus the 13-valent pneumococcal vaccine in healthy infants and toddlers.

**Data source:**

We searched PubMed, Scopus, Embase, and Cochrane on January 20, 2026, for randomized controlled trials comparing the 20-valent pneumococcal conjugate vaccine with the 13-valent pneumococcal conjugate vaccine in healthy infants and toddlers (0-3 years). We computed risk ratios with 95% confidence intervals for all outcomes, using a random-effects model. Heterogeneity was assessed using I^2^ statistics.

**Data synthesis:**

Five randomized clinical trials were included, comprising 5,604 patients, of whom 3,059 received the 20-valent pneumococcal conjugate vaccine. There were no significant differences between the 20-valent pneumococcal conjugate vaccine and the 13-valent pneumococcal conjugate vaccine in adverse event incidence after doses 1-3 (risk ratio 1.00; 95%CI 0.92–1.10; p=0.952; I^2^=0%), or after dose 4 (risk ratio 0.90; 95%CI 0.76–1.07; p=0.238; I^2^=36%). Similarly, no significant differences were observed in serious adverse events, fever, irritability, and injection site pain. The 20-valent pneumococcal conjugate vaccine was associated with a higher incidence of decreased appetite after doses 2 (risk ratio 1.13, 95%CI 1.02–1.24; p=0.019) and 3 (risk ratio 1.34, 95%CI 1.14–1.58; p<0.001). In contrast, a significantly lower risk of local redness was observed with the 20-valent pneumococcal conjugate vaccine after dose 2 (risk ratio 0.93; 95%CI 0.86–0.99; p=0.034).

**Conclusions:**

The 20-valent pneumococcal conjugate vaccine demonstrated a safety profile largely comparable to the 13-valent pneumococcal conjugate vaccine, supporting its use in routine pediatric immunization programs.

## RESUMO

**Objetivo:**

Revisão sistemática e metanálise para comparar a segurança da vacina pneumocócica conjugada 20-valente (VPC20) com a VPC13 em lactentes e crianças menores de três anos.

**Fontes de dados:**

Realizou-se busca sistemática nas bases PubMed, Scopus, Embase e Cochrane até 20 de janeiro de 2026. Foram incluídos ensaios clínicos randomizados (ECRs) que compararam VPC20 e VPC13 em crianças saudáveis de zero a três anos. Estimaram-se riscos relativos (RR) e intervalos de confiança de 95% (IC95%) por modelo de efeitos aleatórios, com avaliação da heterogeneidade pelo índice 12.

**Síntese dos dados:**

Foram incluídos cinco ECRs, totalizando 5.604 pacientes, dos quais 3.059 receberam VPC20. Não foram observadas diferenças estatisticamente significativas na ocorrência de eventos adversos totais após as doses 1–3 (RR 1,00; IC95% 0,92–1,10; I^2^=0%) ou após a dose 4 (RR 0,90; IC95% 0,76–1,07; I^2^=36%). De forma similar, não foram identificadas diferenças significativas na ocorrência de eventos adversos graves, febre, irritabilidade ou dor no local da injeção. A VPC20 associou-se a maior risco de diminuição do apetite após as doses 2 (RR 1,13; IC95% 1,02–1,24) e 3 (RR 1,34; IC95% 1,14–1,58), e a menor risco de vermelhidão local após a dose 2 (RR 0,93; IC95% 0,86–0,99).

**Conclusões:**

A VPC20 apresentou perfil de segurança comparável ao da VPC13 em lactentes e crianças pequenas, sustentando seu uso na imunização pediátrica de rotina.

## INTRODUCTION

*Streptococcus pneumoniae* remains a leading cause of morbidity and mortality globally in children under five years of age.^1^Available pneumococcal conjugate vaccines (PCVs) for pediatric use include the 10-valent, 13-valent (PCV13), 15-valent (PCV15), and 20-valent (PCV20) formulations.^2,3^ PCV13 has substantially reduced the burden of invasive pneumococcal disease.^4^ However,serotypes not included in PCV13 (8, 10A, 11A, 12F, 15B, 22F, and 33F) continue to account for a meaningful proportion of residual disease.^5,6^ PCV20, first licensed for pediatric use in 2023, adds seven epidemiologically relevant serotypes to PCV13.^7^ Althoughphase 2/3 randomized controlled trials (RCTs) established PCV20 immunogenicity in infants and toddlers,^8,9,10,11,12^ the comparative safety between PCV20 and PCV13 remains limited by the statistical power of individual trials, particularly for rare but clinically important adverse events (AEs).

PCV13 is a good comparator for three main reasons:

1. It has been the global pediatric standard of care since 2010,^13^ with extensive safety and efficacy data before PCV20 ’s approval for pediatric use^14^ and served as the reference vaccine when pivotal PCV20 trials were designed in 2020;^10,12^
2. US Food and Drug Administration (FDA) approval of PCV20 was based on non-inferiority comparisons against PCV13,^14,15^ the required benchmark for demonstrating comparable safety and immunogenicity to the current standard of care;
3. Although PCV15 was approved for pediatric use in 2022, it was unavailable when the PCV20 pediatric trials were designed and initiated; moreover, PCV15 has limited pediatric safety data compared to PCV13.^3^

Therefore, a meta-analysis comparing PCV20 with PCV13 provides the most clinically relevant and statistically powerful safety comparison, leveraging PCV13 ’s extensive post-marketing experience to contextualize PCV20 safety findings. Current guidelines still allow the use of PCV13, although PCV15 or PCV20 are preferred, confirming that PCV13 remains an acceptable standard of care.^16^

Therefore, given the critical importance of safety data in guiding clinical practice and public health immunization policies, a systematic synthesis of evidence is warranted. Aggregating safety outcomes across RCTs can provide a more comprehensive assessment of AEs, inform risk-benefit evaluations, and support decision-making by clinicians, caregivers, and policymakers. Thus, the primary objective of this systematic review and meta-analysis was to compare the safety profile of PCV20 versus PCV13 in healthy infants and toddlers (<3 years) using evidence from RCTs.

## METHOD

This systematic review and meta-analysis was conducted in accordance with the Cochrane Handbook for Systematic Reviews of Interventions^17^ and reported according to the Preferred Reporting Items for Systematic Reviews and Meta-Analyses (PRISMA) 2020 statement.^18^ The prospective protocol was registered on PROSPERO (CRD420251114554).

Inclusion in this meta-analysis was restricted to studies that met the following eligibility criteria:

1. Healthy infants and toddlers (<3 years);
2. Studies comparing the administration of PCV20 with PCV13 with any dosing schedule (e.g., 3+1, 2+1, 3+0);
3. Co-administration with routine childhood vaccines permitted;
4. RCTs;
5. Reported at least one clinical outcome of interest.

We excluded studies:

1. That included patients with underlying chronic conditions, immunocompromised status, or age >3 years;
2. That evaluated PCV20 in immunocompromised populations or in children with significant comorbidities;
3. That included vaccines other than PCV20 as the primary intervention;
4. That were observational studies, case reports, or non-randomized trials.

Regarding data sources, we systematically searched PubMed, Scopus, Embase, and Cochrane Central Register of Controlled Trials from inception to January 20, 2026. The complete search strategy for each database is available in Table 1. No language restrictions were applied.

**Table 1:** Complete search strategies for each database.

Database	Search strategy	Records retrieved
Cochrane	(“20-valent Pneumococcal Conjugate Vaccine” OR PCV20 OR PCV-20) AND (PCV13 OR “pneumococcal 13-valent conjugate vaccine” OR “13-valent Pneumococcal Conjugate Vaccine” OR 13vPCV OR “Pneumococcal conjugate vaccine 13-valent”) AND (infant OR toddler OR child OR children OR pediatric OR paediatric OR “preschool-aged children” OR “early childhood”)	17
Scopus	(“20-valent Pneumococcal Conjugate Vaccine” OR PCV20 OR PCV-20) AND (PCV13 OR “pneumococcal 13-valent conjugate vaccine” OR “13-valent Pneumococcal Conjugate Vaccine” OR 13vPCV OR “Pneumococcal conjugate vaccine 13-valent”) AND (infant OR toddler OR child OR children OR pediatric OR paediatric OR “preschool-aged children” OR “early childhood”)	147
PubMed	(“20-valent Pneumococcal Conjugate Vaccine” OR PCV20 OR PCV-20) AND (PCV13 OR “pneumococcal 13-valent conjugate vaccine” OR “13-valent Pneumococcal Conjugate Vaccine” OR 13vPCV OR “Pneumococcal conjugate vaccine 13-valent” OR “Pneumococcal Vaccines” [MeSH]) AND (infant OR toddler OR child OR children OR pediatric OR paediatric OR “preschool-aged children” OR “early childhood” OR “Infant” [MeSH] OR “Child, Preschool” [MeSH])	197
Embase	(‘20-valent Pneumococcal Conjugate Vaccine ’ OR PCV20 OR PCV-20 OR ‘pneumococcal 20 valent conjugate vaccine ’/exp) AND (PCV13 OR ‘pneumococcal 13-valent conjugate vaccine ’ OR ‘13-valent Pneumococcal Conjugate Vaccine ’ OR 13vPCV OR ‘Pneumococcal conjugate vaccine 13-valent ’ OR ‘pneumococcal 13 valent conjugate vaccine ’/exp) AND (infant OR toddler OR child OR children OR pediatric OR paediatric OR children OR ‘preschool-aged children ’ OR ‘early childhood ’ OR ‘child ’/exp OR ‘infant ’/exp)	299

Source: Search strategies developed by the authors for this systematic review.

Records were screened independently by two authors (H.U. and E.S.), followed by full-text assessment. Data from included studies were extracted independently by three authors (H.U., M.B., E.C.) using a standardized data collection form. The extracted variables included study characteristics, participant demographics and baseline characteristics, intervention details (vaccine type, route of administration, vaccination schedule, and concomitant vaccines), safety outcomes, newly diagnosed medical conditions (NDMCs), and the timing of outcome assessment after each vaccine dose and throughout the study period. For studies that presented data exclusively in figures, numerical data were extracted using WebPlotDigitizer (version 5.0). All required data were clearlyreported. Disagreements were resolved by consensus.

Primary safety outcomes were any AE and serious adverse events (SAEs), analyzed separately for the primary vaccination series (doses 1–3) and for the booster dose (dose 4), when data were available. Serious AEs were defined according to the protocol of each trial and applicable regulatory standards; however, specific coding systems and attribution criteria were not uniformly reported across studies.

Secondary outcomes included solicited local reactions (injection site pain, redness, and swelling), solicited systemic reactions (fever, irritability, drowsiness, and decreased appetite), and NDMCs. Fever was extracted as reported by each trial.

Risk of bias was assessed using the Cochrane Risk of Bias version 2 (ROB 2) tool.^19^ Two independent authors completed the risk of bias assessment (H.U. and M.B.). Disagreements were resolved by consensus. Funnel plots were not used due to the limited number of studies included in each outcome analysis (<10), as recommended by the Cochrane Handbook.^17^

The certainty of evidence for the primary outcomes was evaluated using the Grading of Recommendations Assessment, Development, and Evaluation (GRADE) framework, considering risk of bias, inconsistency, indirectness, imprecision, and publication bias. Certainty ratings (high, moderate, low, or very low) were assigned based on these domains, and Summary of Findings tables were generated to provide transparent grading of effect estimates.

Risk ratios (RR) with 95% confidence intervals (CIs) were used to compare treatment effects for categorical endpoints, with statistical significance defined as a two-sided p<0.05. We assessed heterogeneity with I^2^ statistics and Cochran Q test; p-values <0.10 and I^2^>25% were considered significant for heterogeneity. DerSimonian and Laird random-effects model was used^20^ and statistical analyses were performed using R software v4.5.1.

To evaluate robustness, we performed two sensitivity analyses:

1. Leave-one-out analysis, systematically removing each study; and
2. Comparison of the random-effects model with a common-effect model.

## RESULTS

The systematic search yielded 660 records. After removing duplicates and applying eligibility criteria, six studies were selected for full-text review (Figure 1). One study was excluded after full-text review because it evaluated PCV20 solely as a booster dose in children previously vaccinated with PCV13, whereas this review was restricted to studies directly comparing PCV13 and PCV20 as distinct vaccination groups. Five studies met the criteria and were included in the meta-analysis.

**Figure 1:**
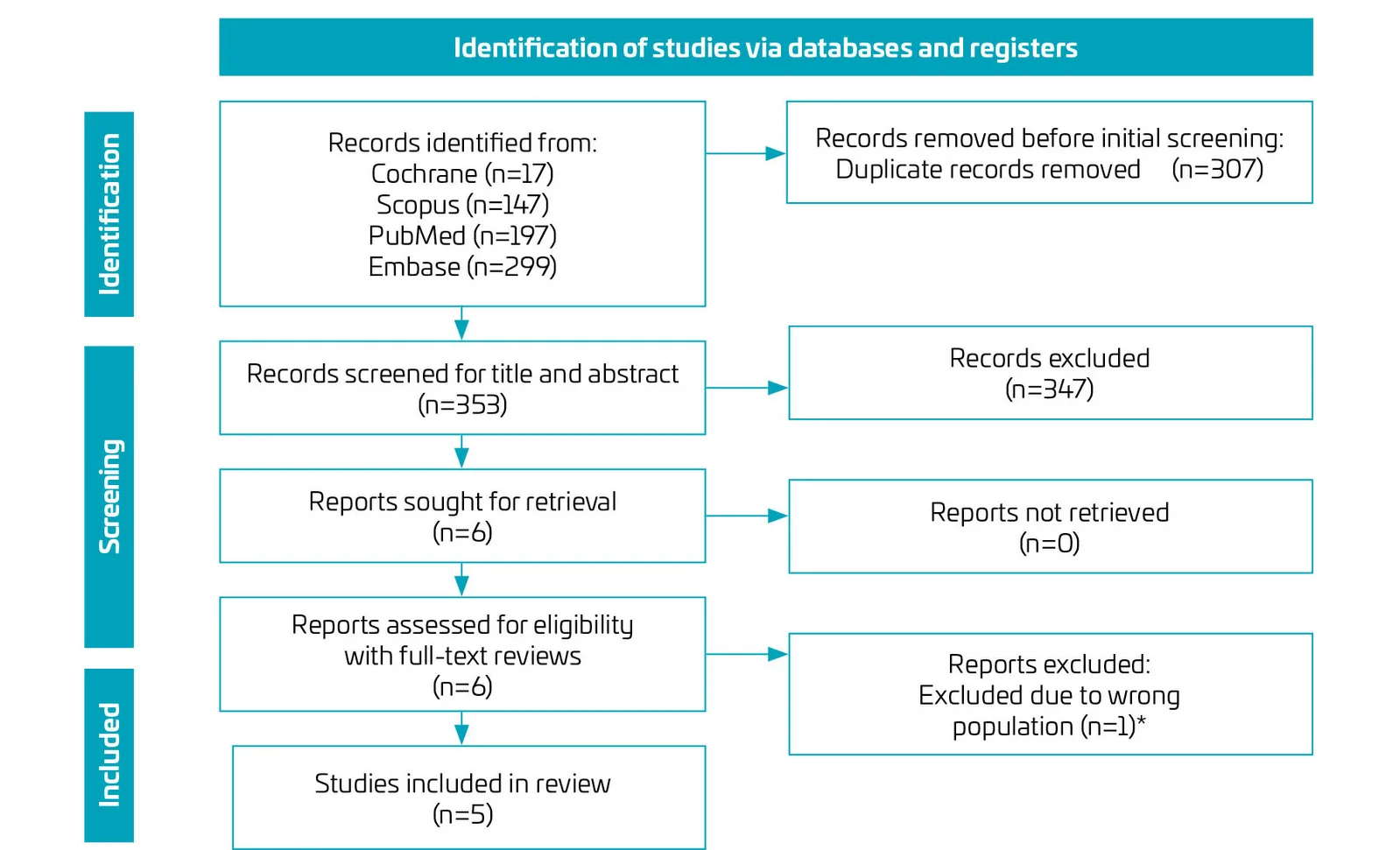
PRISMA flow diagram of the study selection process comparing the safety of the 20-valent versus the 13-valent pneumococcal conjugate vaccines in infants and toddlers.

Source: Page et al.^19^

*The study evaluated PCV20 as a booster in children who had already received PCV13, while the review included studies comparing PCV13 versus PCV20 as distinct vaccination groups.

PCV20: 20-valent pneumococcal conjugate vaccine; PCV13: 13-valent pneumococcal conjugate vaccine;

A total of 5,604 children were included, of whom 3,059 (~55.6%) received PCV20. Baseline characteristics were comparable (Table 2). In all trials, pneumococcal vaccines were co-administered with routine childhood vaccines according to local immunization schedules. Regarding gestational age, most trials included infants born at >36 weeks; one explicitly included late-preterm infants (≥34 to <37 weeks), with safety comparable to that of term infants.^9^

**Table 2:** Characteristics of the studies comparing the safety profile of the 20-valent versus the 13-valent pneumococcal conjugate vaccines in infants and toddlers.

	Senders et al.^9^ (n=460)		Senders et al.^13^ (n=1988)		Korbal et al.^11^ (n=1204)		Hajdu et al.^10^ (n=1503)		Ishihara et al.^12^ (n=449)	
Characteristics	PCV 20(n=232)	PCV 13(n=228)	PCV 20(n=1001)	PCV 13(n=987)	PCV 20(n=601)	PCV 13(n=603)	PCV 20(n=1000)	PCV 13 (n=503)	PCV 20(n=225)	PCV 13(n=224)
Study design	RCT		RCT		RCT		RCT		RCT	
Country	Single-country^a^		Single-country^a^		Multicenter^b^		Multicenter^c^		Single-country^d^	
Population	Healthy infants		Healthy infants		Healthy infants		Healthy infants		Healthy infants	
Route of Administration	IM (0.5mL)		IM (0.5mL)		IM (0.5mL)		IM (0.5mL)		SC (0.5mL)	
Vaccination Schedule	4-dose series		4-dose series		3-dose series		4-dose series		4-dose series	
Concomitant Vaccines^e^	Routine vaccines^f^		Routine vaccines^g^		Routine vaccines^h^		Routine vaccines^i^		Routine vaccines^j^	
Age^k^	64.5±8.1	64.5±6.7	65.9±8.0	65.6±7.1	69.2±17.8	69.7±18.3	64.6±8.5	65.0±9.0	72.0±10.0^l^	72.0±12.0^l^
Male, %	51.7	49.6	51.7	51.2	49.8	51.6	51.7	48.5	48.0	51.2
White race, %	69.4	75.0	75.2	75.3	97.3	98.2	86.8	88.5	0.0	0.0

PCV20: 20-valent pneumococcal conjugate vaccine; PCV13: 13-valent pneumococcal conjugate vaccine; RCT: Randomized controlled trial; IM: Intramuscular; SC: Subcutaneous.

Notes: Single-country^a^: United States of America;

Multicenter^b^: Australia, Belgium, Czech Republic, Denmark, Estonia, Finland, Italy, Netherlands, Norway, Poland, Slovakia;

Multicenter^c^: Argentina, Canada, Chile, Czech Republic, Finland, Germany, Greece, Hungary, Spain, United States, and Puerto Rico;

Single-country^d^

Japan;Concomitant vaccines^e^: concomitant vaccines were administered according to local routine immunization schedules, as detailed in references f, g, h, i and j;

Routine vaccines^f^: DTaP–HepB–IPV, *Haemophilus influenzae* type b (Hib), and rotavirus vaccines (Doses 1–3); measles–mumps–rubella (MMR) vaccine (Dose 4); influenza vaccine (administered to age-eligible participants during the influenza season);

Routine vaccines^g^: DTaP–HepB–IPV, Hib, and rotavirus vaccines (Doses 1–3); MMR and varicella vaccines (Dose 4);

Routine vaccines^h^: Infanrix Hexa (DTaP–HepB–IPV–Hib) at all doses; MMR and varicella vaccines (Dose 3); rotavirus and influenza vaccines (per local guidelines);

Routine vaccines^i^: Routine pediatric vaccines according to local schedules (e.g., DTaP, Hib, polio, MMR, varicella, etc.);

Routine vaccines^j^: *Haemophilus influenzae* type b (Hib) vaccine; pertussis, diphtheria, tetanus, and poliomyelitis combination vaccine; and varicella vaccine;

Age^k^: mean±Standard Deviation, days, at dose 1;

ISHIHARA, 2024^l^: Data for age were originally given in months (2.4±0.40 and 2.4±0.33). It was converted to days by multiplying by 30.4 (the average number of days in a month

Source: Authors’ elaboration based on data extracted from the included studies. Hajdu et al.^9^, Ishihara et al.^11^, Korbal et al.^10^, Senders et al.^8^ and Senders et al.^12^

Regarding the primary safety outcomes, there were no significant differences between PCV20 and PCV13 for any AE following doses 1 to 3 (Figure 2) or dose 4 (Table 3; p=0.238; I^2^=36.0%). Similarly, no significant differences were observed between PCV20 and PCV13 for SAEs following doses 1 to 3 (Table 3; p=0.903; I^2^=0.0%) or dose 4 (Table 3; p=0.603; I^2^=0.0%).

**Figure 2:**
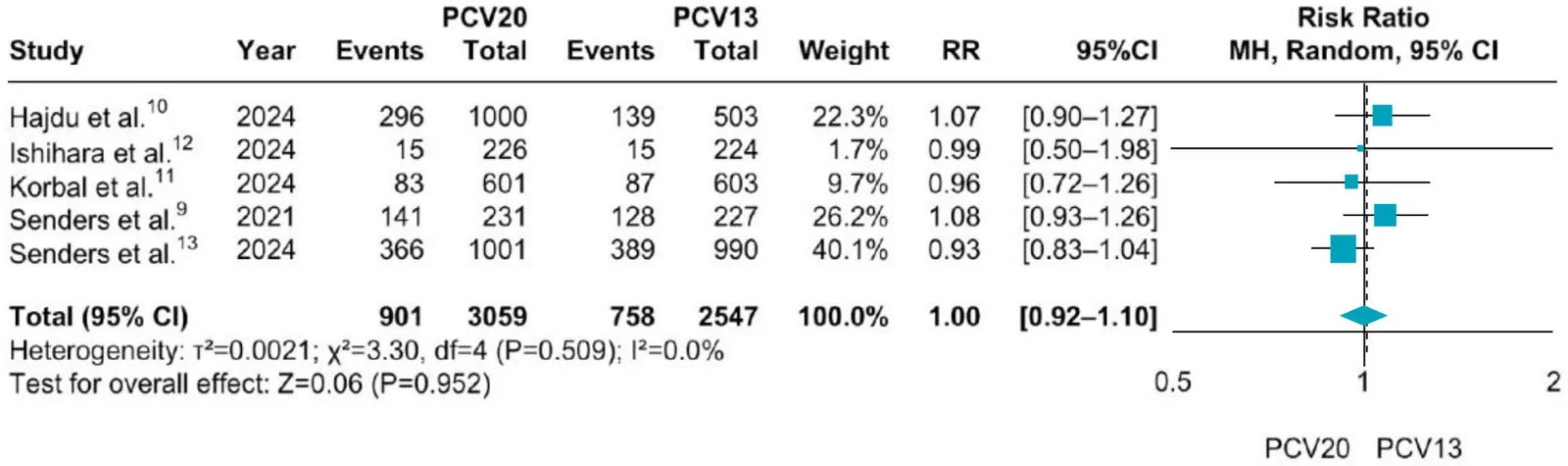
Forest plot comparing the risk of any adverse event following doses 1 to 3 of the 20-valent versus the 13-valent pneumococcal conjugate vaccines in infants and toddlers.

Source: Authors’ elaboration based on data from Hajdu et al.^9^, Ishihara et al.^11^, Korbal et al.^10^, Senders et al.^8^, and Senders et al.^12^

**Table 3:** Summary of findings and certainty of evidence (GRADE) for safety outcomes comparing the 20-valent versus the 13-valent pneumococcal conjugate vaccines in infants and toddlers.

Anticipated absolute effects					
	Number of participants (studies)	Relative effect (95%CI)	Certainty of evidence (GRADE)	Risk with [PCV13]	Risk difference with [PCV20]
Any AE (D1–D3)	5606 (5 RCTs)	RR 1.00 (0.92 to 1.10)	⨁⨁⨁⨁ High	298 per 1,000	0 fewer per 1,000 (24 fewer to 30 more)
Any AE (D4)	3904 (4 RCTs)	RR 0.90 (0.76 to 1.07)	⨁⨁⨁⨁ High^a^	155 per 1,000	15 fewer per 1,000 (37 fewer to 11 more)
SAE (all doses)	5152 (4 RCTs)	RR 1.09 (0.74 to 1.60)	⨁◯◯◯ Very low^b,c^	45 per 1,000	4 more per 1,000 (12 fewer to 27 more)
SAE (D1–D3)	4698 (3 RCTs)	RR 0.96 (0.47 to 1.95)	⨁⨁◯◯ Low^d^	7 per 1,000	0 fewer per 1,000 (4 fewer to 6 more)
SAE (D4)	3078 (2 RCTs)	RR 1.33 (0.45 to 3.90)	⨁⨁◯◯ Low^e^	4 per 1,000	1 more per 1,000 (2 fewer to 11 more)
Decreased appetite D3	5607 (5 RCTs)	RR 1.34 (1.14 to 1.58)	⨁⨁⨁◯ Moderate^f^	190 per 1,000	65 more per 1,000 (27 more to 110 more)
Irritability D2	5451 (5 RCTs)	RR 0.98 (0.91 to 1.05)	⨁⨁⨁◯ Moderate^g,h^	665 per 1,000	13 fewer per 1,000 (60 fewer to 33 more)
Fever D1	5607 (5 RCTs)	RR 1.12 (0.88 to 1.42)	⨁⨁⨁◯ Moderate^i,j^	86 per 1,000	10 more per 1,000 (10 fewer to 36 more)
Fever D2	5451 (5 RCTs)	RR 1.06 (0.88 to 1.28)	⨁⨁⨁◯ Moderate^k,l^	156 per 1,000	9 more per 1,000 (19 fewer to 44 more)
Redness D2	5607 (5 RCTs)	RR 0.93 (0.86 to 0.99)	⨁⨁⨁⨁ High	316 per 1,000	22 fewer per 1,000 (44 fewer to 3 fewer)

CI: confidence interval; GRADE: Grading of Recommendations Assessment, Development and Evaluation; PCV20: 20-valent pneumococcal conjugate vaccine; PCV13: 13-valent pneumococcal conjugate vaccine; AE = adverse event; RCT: Randomized controlled trial; RR: risk ratio; SAE: serious adverse event; D1–D3: doses 1 through 3; D4: booster dose.

^*^The risk in the intervention group (and its 95% confidence interval) is based on the assumed risk in the comparison group and the relative effect of the intervention (and its 95% confidence interval).

**Explanations:**

**^a.^** No downgrade as the 95%CI (0.76–1.07) crosses the line of no effect but remains within the clinically neutral range (0.75–1.25), indicating small and likely irrelevant differences; total events exceeded the optimal information size (≥300).

**^b.^** Downgraded one level for inconsistency due to moderate heterogeneity (I2 = 54%) and variability in study results.

**^c.^** Downgraded two levels because the 95%CI (0.74–1.60) is wide, crosses the line of no effect, and includes both appreciable benefit and harm; total events (<300) did not meet the optimal information size.

**^d.^** Downgraded two levels because the 95%CI (0.47–1.95) is extremely wide, crossing the line of no effect and including both substantial benefit and harm; total events (<100) indicate insufficient information size.

**^e.^** The confidence interval is extremely wide, including large benefit and substantial harm; the evidence is highly imprecise.

**^f.^** Downgraded one level for inconsistency due to moderate heterogeneity (I2 = 60.5%) (p = 0.0386).

**^g.^** Downgraded one level for inconsistency due to moderate heterogeneity (I2 = 66.7%) (p=0.503).

**^h.^** No downgrade as the 95%CI (0.91–1.05) is entirely within the clinically neutral range (0.75–1.25), indicating small and likely irrelevant differences; total events exceeded the optimal information size (≥300).

**^i.^** No downgrade as heterogeneity was moderate (I2=47.5%) but not statistically significant, and study estimates were consistent in direction of effect.

**^j.^** Downgraded one level because the 95%CI crosses the line of no effect and extends slightly beyond the clinically neutral limit (>1.25), suggesting a possible increase in risk. The total number of events was adequate (>300).

**^k.^** No downgrade as heterogeneity was moderate (I2≈50%) but not statistically significant, and study estimates were consistent in direction of effect.

**^l.^** Downgraded one level because the 95%CI crosses the line of no effect and slightly exceeds the clinically neutral limit (>1.25), suggesting a potential increase in risk. The total number of events was adequate (>300).

Source: Authors’ elaboration based on data from the included studies and the GRADE tool

Regarding local reactions, there were no significant differences between groups in injection site pain following dose 1 (RR 1.02; 95%CI 0.95–1.10; p=0.538), dose 2 (RR 0.98; 95%CI 0.91– 1.06; p=0.655), dose 3 (RR 1.01; 95%CI 0.94–1.08; p=0.860), or dose 4 (RR 0.99; 95%CI 0.90–1.08; p=0.776), all with I^2^=0.0%. No significant differences were observed for redness following dose 1 (RR 1.01; 95%CI 0.94–1.09; p=0.713; I^2^=0.0%), dose 3 (RR 0.99; 95%CI 0.90–1.09; p=0.869; I^2^=42.1%), and dose 4 (RR 0.94; 95%CI 0.85–1.04; p=0.227; I^2^=32.8%). However, after dose 2, the risk of redness was significantly lower with PCV20 compared with PCV13 (Table 3; p=0.034; I^2^=10.1%). No significant differences between the two vaccines were identified for swelling across the four-dose series, with pooled RRs of 1.00 (95%CI 0.89–1.12; p=0.976; I^2^=27.9%) following dose 1, 0.98 (95%CI 0.90–1.06; p=0.554; I^2^=0.0%) following dose 2, 0.99 (95%CI 0.88–1.11; p=0.862; I^2^=33.2%) following dose 3, and 1.02 (95%CI 0.94–1.10; p=0.700; I^2^=2.6%) following dose 4.

Regarding systemic reactions, there were no significant differences between PCV20 and PCV13 for decreased appetite following dose 1 (RR 0.99; 95%CI 0.90–1.09; p=0.890; I^2^=39.4%) or dose 4 (RR 1.00; 95%CI 0.90–1.11; p=0.964; I^2^=0.0%). However, there was a higher incidence of decreased appetite in children who received PCV20 compared to PCV13 following dose 2 (RR 1.13; 95%CI 1.02–1.24; p=0.019; I^2^=28.3%) and dose 3 (Table 3; p<0.001; I^2^=60.5%). Similarly, no significant differences were observed between the two vaccines for drowsiness following dose 1 (RR 1.00; 95%CI 0.96–1.04; p=0.990; I^2^=3.2%), dose 2(RR 0.97; 95%CI 0.93–1.03; p=0.330; I^2^=0.0%), dose 3 (RR 0.99; 95%CI 0.93–1.05; p=0.675; I^2^ = 10.6%), or dose 4 (RR 0.98; 95%CI 0.90–1.07; p=0.695; I^2^=0.0%). No significant differences were identified for fever following dose 1 (Table 3; p=0.346; I^2^=47,5%), dose 2 (Table 3; p=0.539; I^2^=49.9%), dose 3 (RR 0.98; 95%CI 0.86–1.11; p=0.726; I^2^=0.0%), or dose 4 (RR 1.05; 95%CI 0.92–1.20; p=0.456; I^2^=0.0%). Likewise, no significant differences were observed for irritability following dose 1 (RR 0.99; 95%CI 0.96–1.03; p=0.760; I^2^=0.0%), dose 2 (Table 3; p=0.503; I^2^=66.7%), dose 3 (RR 1.01; 95%CI 0.97– 1.06; p=0.521; I^2^=0.0%), or dose 4 (RR 0.99; 95%CI 0.94–1.05; p=0.796; I^2^=0.0%).

Finally, regarding NDMCs, no significant difference was found between PCV13 and PCV20 (RR 0.95; 95%CI 0.72–1.25; p=0.717; I^2^=0.0%). In the trial by Hajdu et al.,^9^ in which NDMCs were defined as newly identified diseases or medical conditions expected to be persistent or long-lasting, the more commonly reported cases were dermatitis, eczema, or food and milk allergies.

Leave-one-out sensitivity analyses for the outcomes of any AEs and SAEs revealed consistent results after omitting each individual study. The estimates for any AEs were stable across doses 1–3, while results for dose 4 varied but remained non-significant. Similarly, analyses for SAEs showed stable, non-significant effect estimates across doses 1–3; for SAEs after dose 4, however, the analysis included only two studies, limiting the interpretability of the leave-one-out approach. Leave-one-out sensitivity analyses were also performed for decreased appetite after dose 3, fever after dose 1, fever after dose 2, irritability after dose 2, and redness after dose 2, outcomes with statistical significance and/or high heterogeneity, with consistent results across all omissions.

Random vs. common model sensitivity analyses were performed for any AEs across doses 1-3, any AEs after dose 4, SAEs across doses 1-3, SAEs after dose 4, decreased appetite after dose 3, fever after dose 1, fever after dose 2, irritability after dose 2, and redness after dose 2. Across all analyses, the choice of statistical model did not materially affect the direction, magnitude, or significance of the pooled estimates. Heterogeneity was low to moderate (I^2^ ranging from 0 to 66.7%), and all results remained robust, supporting the reliability of our main findings.

The risk-of-bias assessment indicated an overall low risk across all included studies, as reported in Table 4. Using the GRADE framework, certainty of evidence was rated as high for the primary outcome of any AE following the primary series (doses 1–3) and after the booster dose, supported by consistent effect estimates and low risk of bias across studies. Certainty of evidence was rated as moderate for most selected solicited local and systemic reactions, with downgrades occurring in selected dose-specific analyses due to heterogeneity or imprecision. In contrast, the certainty of evidence for SAEs across all doses was rated as low to very low, reflecting wide CIs and a limited number of events across dose-specific analyses, which limits the ability to detect differences in rare outcomes. Evidence certainty is summarized in Table 3.

**Table 4:** Risk of bias assessment using the Cochrane RoB 2 tool.

Study	D1	D2	D3	D4	D5	Overall
Hajdu et al.^10^	Low	Low	Low	Low	Low	Low
Ishihara et al.^12^	Low	Low	Low	Low	Low	Low
Korbal et al.^11^	Low	Low	Low	Low	Low	Low
Senders et al.^9^	Low	Low	Low	Low	Low	Low
Senders et al.^13^	Low	Low	Low	Low	Low	Low

Definitions of the RoB 2 domains: D1: Bias arising from the randomization process; D2: Bias due to deviations from the intended intervention; D3: Bias due to missing outcome data; D4: Bias in measurement of the outcome; D5: Bias in selection of the reported result.

Source: Authors ’ elaboration based on the Cochrane RoB 2 tool.

## DISCUSSION

This systematic review and meta-analysis of five RCTs compares the safety of PCV20 versus PCV13 in healthy infants and toddlers. PCV20 demonstrated a safety profile largely comparable to the well-established PCV13 for most assessed AEs, including solicited local and systemic reactions and any AEs throughout the pneumococcal vaccination series. This finding is relevant and supports PCV20 introduction in immunization programs for broader serotype coverage. While no statistically significant differences in SAEs were observed, the low incidence of these outcomes limits the ability to draw definitive conclusions.

A notable finding in our analysis was a statistically significant increase in the risk of decreased appetite after the second and third PCV20 doses. This pattern was also observed in the pivotal trials included in this review,^9,10^ which reported a higher incidence of decreased appetite following doses 2 and 3 in the PCV20 group compared with PCV13. Importantly, the included trials did not report associated clinical sequelae, such as weight loss. This may inform caregiver counseling during the primary series.

For other specific AEs, such as drowsiness, fever, injection site pain, and swelling, the results further reinforced the overall comparability of the safety profiles. A transient variation was observed for redness, with PCV20 showing a lower risk after dose 2. No significant differences were found for irritability across the series. The substantial heterogeneity observed for this symptom after dose 2 (I^2^=66.7%) is plausibly explained by contextual differences across studies — including variation in study settings, populations, and parental reporting methods — given that irritability is a highly subjective outcome. These contextual differences were not expected to influence biological reactogenicity but may affect reporting patterns.

Our findings consolidate the evidence from Phase 3 studies^8,9^on pneumococcal conjugate vaccines and their safety. Earlier formulations such as heptavalent (PCV7) and PCV13 were shown to be both effective and well tolerated in large clinical trials.^5,21,22^PCV20 development was driven by the continued global burden of *Streptococcus pneumoniae* among children under five years of age^1,23^ and by the appearance of additional invasive serotypes following widespread PCV13 use.^6,24^ Importantly, PCV13 was the established standard when PCV20 trials^9,10^ were designed and is one of the best-characterized pneumococcal vaccines in terms of safety.^25^ Therefore, demonstrating a comparable safety profile relative to PCV13 provides a clinically meaningful benchmark for interpreting the safety profile of PCV20. Our meta-analysis adds to this evidence base, showing that the inclusion of seven additional serotypes in PCV20 does not appear to compromise short-term safety outcomes when compared with PCV13.

Real-world surveillance further supports the safety of PCV20. In USadults, post-licensure data showed a profile consistent with clinical trials, consisting mostly of non-serious local and systemic reactions.^26^ While a potential signal for Guillain-Barré Syndrome in the adult population is under investigation by the Centers for Disease Control (CDC) and FDA, no other unexpected concerns have emerged.^26^ Although direct real-world data for PCV20 in infants are still accumulating following its 2023 approval, the extensive safety record of PCV13 provides a robust benchmark.^8,9^ Large-scale surveillance of PCV13 in children found no increased risk for serious events like febrile seizures or anaphylaxis, reinforcing the reliability of this vaccine platform in pediatric populations.^25^

Future research should prioritize:

1. Adequately powered studies for rare AEs and longer follow-up for late-onset outcomes;
2. Evaluating diverse pediatric populations (preterm, chronic conditions, immunocompromised);
3. Harmonizing solicited AE definitions and reporting; and
4. Head-to-head comparisons across different schedules and co-administration patterns.

Linking trial data with real-world electronic health records/safety monitoring can accelerate rare signal detection.

In addition to clinical safety, economic considerations also influence PCV20 adoption. Although PCV20 may have higher acquisition costs, its broader serotype coverage could reduce pneumococcal disease burden, including invasive disease and hospitalizations.^1,2^ However, initial financial impacts may represent a challenge, particularly in low- and middle-income settings. Formal cost-effectiveness analyses were beyond the scope of this review, and context-specific evaluations are needed to support policy decision-making.

This study has several limitations that should be acknowledged. First, the limited number of RCTs (n=5) included constrained the feasibility of predefined subgroup analyses, precluded formal assessment of publication bias, and reduced statistical power to detect very rare AEs and SAEs. Second, variability in vaccination protocols across the trials included may have influenced the pooled estimates. Studies differedwith respect to the route of vaccine administration (intramuscular or subcutaneous) and the vaccination schedule (three-dose primary series or four-dose regimen including a booster dose). This may have contributed to clinical heterogeneity, though analyses were performed separately for doses 1–3 and dose 4 whenever possible. Third, moderate to substantial heterogeneity was observed for certain subjective outcomes, particularly irritability after dose 2 (I^2^=66.7%), likely reflecting differences in parental reporting and outcome ascertainment across trials rather than true biological differences between vaccines. To address this, sensitivity analyses were performed and demonstrated the robustness of the pooled estimates. Fourth, follow-up durations were restricted to the trial periods and therefore do not capture potential long-term or delayed AEs. Fifth, all trials co-administered PCV20 with routine childhood vaccines according to local immunization schedules (Table 2) and, to our knowledge, no RCTs have evaluated the safety of PCV20 as a standalone intervention in infants and toddlers. As a result, it is not possible to isolate AEs attributable solely to the pneumococcal component or to directly compare isolated versus concomitant administration. This represents an important limitation of the current evidence base and highlights the need for studies specifically designed to evaluate the independent safety profile of PCV20. Sixth, data on very preterm infants (<32 weeks) are scarce, as pivotal RCTs primarily enrolled infants born at >36 weeks of gestation, limiting generalizability to more immature populations. Finally, data on parental or healthcare provider acceptability, which may influence vaccine uptake and real-world implementation, were not systematically reported and could not be evaluated in this meta-analysis.

Overall, PCV20 demonstrated a safety profile comparable to PCV13 across local and systemic AEs in RCTs, with no statistically significant differences observed. While no differences in SAEs were identified, conclusions regarding rare SAEs should be interpreted with caution due to limited statistical power. These findings support policy decisions to introduce PCV20 into national immunization schedules for infants and toddlers, alongside ongoing post-marketing surveillance.

## Data Availability

The database that originated the article is available with the corresponding author.
